# Primary tumor surgery improves survival in non-metastatic primary urethral carcinoma patients: a large population-based investigation

**DOI:** 10.1186/s12885-021-08603-z

**Published:** 2021-07-27

**Authors:** Jie Wu, Yu-Chen Wang, Wen-Jie Luo, Bo-Dai, Ding-Wei Ye, Yi-Ping Zhu

**Affiliations:** 1 Department of Urology, Fudan University Shanghai Cancer Center, No. 270 Dong an Road, Shanghai, 200032 People’s Republic of China; 2 Department of Oncology, Shanghai Medical College, Fudan University, Shanghai, China

**Keywords:** Primary urethral carcinoma, Survival, SEER, Surgery

## Abstract

**Background:**

Primary urethral carcinoma (PUC) is a rare genitourinary malignancy with a relatively poor prognosis. The aim of this study was to examine the impact of surgery on survival of patients diagnosed with PUC.

**Methods:**

A total of 1544 PUC patients diagnosed between 2004 and 2016 were identified based on the SEER database. The Kaplan-Meier estimate and the Fine and Gray competing risks analysis were performed to assess overall survival (OS) and cancer-specific mortality (CSM). The multivariate Cox regression model and competing risks regression model were used to identify independent risk factors of OS and cancer-specific survival (CSS).

**Results:**

The 5-yr OS was significantly better in patients who received either local therapy (39.8%) or radical surgery (44.7%) compared to patients receiving no surgery of the primary site (21.5%) (*p <* 0.001). Both local therapy and radical surgery were each independently associated with decreased CSM, with predicted 5-yr cumulative incidence of 45.4 and 43.3%, respectively, compared to 64.7% for patients receiving no surgery of the primary site (*p <* 0.001). Multivariate analyses demonstrated that primary site surgery was independently associated with better OS (local therapy, *p* = 0.037; radical surgery, *p <* 0.001) and decreased CSM (*p* = 0.003). Similar results were noted regardless of age, sex, T stage, N stage, and AJCC prognostic groups based on subgroup analysis. However, patients with M1 disease who underwent primary site surgery did not exhibit any survival benefit.

**Conclusion:**

Surgery for the primary tumor conferred a survival advantage in non-metastatic PUC patients.

**Supplementary Information:**

The online version contains supplementary material available at 10.1186/s12885-021-08603-z.

## Background

Primary urethral carcinoma (PUC) is a rare genitourinary malignancy with a relatively poor prognosis [1–3]. In 2020, it was estimated that in the United States there are 3970 new diagnoses of cancer of the ureter and other urinary organs, and 1010 will die of these diseases [3]. The 5-yr overall survival rate in PUC patients is reported to be 42% [4, 5]. Disease management of PUC is based on tumor stage, patient sex and tumor location [2, 6, 7]. Surgery, chemotherapy or radiation therapy are standard treatment options for patients diagnosed with PUC [8–10]. Unfortunately, owing to its rare nature, there is a lack of large-scale investigations to support the treatment strategies. The aim of this study was to examine the impact of surgery on survival of patients diagnosed with PUC using a large population-based cancer database.

## Methods

### Selection of patient cohort

We searched Surveillance, Epidemiology, and End Results (SEER) public-access database covering around 27.8% of the U.S. population from 2004 to 2016 and identified patients diagnosed with PUC based on the International Classification of Diseases-O-3 (ICD-O-3) codes C68.0. Only patients who met the following criteria were included: (1) urethra was the primary site; (2) survival time was ≥1 month; and (3) adequate tumor data were available. Data were extracted from the SEER database using SEER*Stat Software (version 8.3.6).

### Data collection and variable definition

Parameters of interest included race, sex, age at diagnosis, the American Joint Commission on Cancer (AJCC) TNM Staging system, histology, tumor size, and grade. Therapy and follow-up information including type of surgical procedure, radiation, chemotherapy, survival months, and vital status were also collected. Surgical codes 30 (Simple/partial surgical removal of primary site), 40 (Total surgical removal of primary site; enucleation), 50 (Surgery stated to be “debulking”), and 60 (Radical surgery) for PUC were merged and collectively defined as “radical surgery”. Transurethral resection and other local tumor destruction or excision procedures (Surgical codes 10 and 20) were merged and collectively defined as “local therapy”. Surgical codes 00 (no surgery of primary site or autopsy only) was defined as “No surgery of primary site”. The overall survival (OS) months for PUC were defined as the time from diagnosis to any cause of death or last follow-up, with patients still alive censored at the last follow-up. For cancer-specific mortality (CSM), deaths not due to PUC were considered as competing risks.

### Statistical analysis

Pearson’s chi-square was applied to compare the distribution of categorical data. Kaplan-Meier survival curves and log-rank tests were utilized to perform survival analysis. The Fine and Gray competing risk analysis was used to evaluate CSM [11, 12]. Multivariate Cox regression and competing risk regression analysis were utilized to identify independent risk factors to predict OS and cancer-specific survival (CSS) of PUC patients. All tests were two sided with a statistical significance set at *p* < 0.05. Statistical analyses were performed using R version 3.5.2 (the R foundation for Statistical Computing, Vienna, Austria).

## Results

### Demographic and clinical characteristics of PUC patients

A total of 1544 PUC patients were identified. The demographic and clinical characteristics of the patient cohort are listed in Table 1. The majority of PUC patients were white (1164, 75.4%), male (971, 62.9%), with stage I (432, 28.2%) or IV (392, 25.6%) TNM stage, and III/ IV grade (833, 54.0%). Among 1544 PUC patients, 642 patients have precise tumor sizes, and the median size was 38.27 mm. Median age at diagnosis was 69.54 years. The pathological types comprised squamous cell carcinoma (437, 28.3%), transitional cell carcinoma (660, 42.7%), adenocarcinoma (252, 16.3%), and other pathological types (195, 12.6%). With regard to therapy, most patients underwent a surgical procedure (1114, 72.2%), and did not receive radiation (1141, 73.9%) or chemotherapy (1067, 69.1%).

**Table 1:** Demographic and clinical characteristics of PUC patients

Variable	Total (*n* = 1544)	Group A: No surgery of primary site (*n* = 403)	Group B: Local therapy (*n* = 532)	Group C: Radical surgery of primary site (*n* = 582)	*p* value (A vs. B)	*p* value (A vs. C)	*p* value (B vs. C)
Age at diagnosis					0.142	<0.001^*^	<0.001^*^
Mean (SD)	69.54 (13.03)	70.4 (13.52)	73.02 (12.85)	65.8 (11.89)			
Race					0.001^*^	0.833	0.003^*^
White	1164 (75.4)	288 (71.5)	431 (81.0)	422 (72.5)			
Black	290 (18.8)	84 (20.8)	81 (15.2)	121 (20.8)			
Other	90 (5.8)	31 (7.7)	20 (3.8)	39 (6.7)			
Sex					<0.001^*^	0.903	<0.001^*^
Male	971 (62.9)	233 (57.8)	385 (72.4)	333 (57.2)			
Female	573 (37.1)	170 (42.2)	147 (27.6)	249 (42.8)			
Grade					<0.001^*^	<0.001^*^	<0.001^*^
I	70 (5.1)	25 (6.2)	27 (5.1)	26 (4.5)			
II	290 (18.8)	70 (17.4)	84 (15.8)	130 (22.3)			
III	457 (29.6)	123 (30.5)	126 (23.7)	203 (34.9)			
IV	376 (24.4)	66 (16.4)	168 (31.6)	139 (23.9)			
Unknown	342 (22.2)	119 (29.5)	127 (23.9)	84 (14.4)			
Histology					<0.001^*^	0.040^*^	<0.001^*^
SCC	437 (28.3)	130 (32.2)	95 (17.9)	205 (35.2)			
TCC	660 (42.7)	146 (36.2)	305 (57.3)	198 (34.0)			
AC	252 (16.3)	59 (14.6)	77 (14.5)	111 (19.1)			
Other	195 (12.6)	68 (16.9)	55 (10.3)	68 (11.7)			
T stage					<0.001^*^	<0.001^*^	<0.001^*^
T0/T1	521 (33.7)	109 (27.0)	274 (51.7)	131 (22.5)			
T2	315 (20.4)	56 (13.9)	110 (20.8)	147 (25.3)			
T3	318 (20.6)	78 (19.4)	54 (10.2)	179 (30.8)			
T4	180 (11.7)	62 (15.4)	35 (6.6)	81 (13.9)			
Tx	207 (13.4)	97 (24.1)	57 (10.8)	44 (7.6)			
N stage					<0.001^*^	<0.001^*^	<0.001^*^
N0	1040 (67.5)	206 (51.2)	416 (78.5)	403 (69.2)			
N1	149 (9.7)	60 (14.9)	23 (4.3)	63 (10.8)			
N2	154 (10.0)	51 (12.7)	33 (6.2)	69 (11.9)			
Nx	198 (12.8)	85 (21.1)	58 (10.9)	47 (8.1)			
M stage					<0.001^*^	<0.001^*^	0.041^*^
M0	1245 (80.8)	258 (64.2)	451 (85.1)	517 (88.8)			
M1	158 (10.3)	96 (23.9)	38 (7.2)	22 (3.8)			
Mx	138 (9.0)	48 (11.9)	41 (7.7)	43 (7.4)			
AJCC stage groups					<0.001^*^	<0.001^*^	<0.001^*^
I	432 (28.2)	68 (16.9)	247 (47.0)	111 (19.2)			
II	228 (14.9)	38 (9.5)	84 (16.0)	104 (18.0)			
III	264 (17.2)	48 (11.9)	42 (8.0)	170 (29.4)			
IV	392 (25.6)	158 (39.3)	84 (16.0)	145 (25.1)			
Unknown	217 (14.2)	90 (22.4)	69 (13.1)	48 (8.3)			
Tumor size, mm (*n* = 642)					<0.001^*^	0.041^*^	0.007^*^
Mean (SD)	38.27 (24.31)	44.30 (24.18)	30.20 (19.82)	38.88 (25.01)			
Radiation					<0.001^*^	<0.001^*^	0.641
Yes	403 (26.1)	146 (36.2)	113 (21.2)	116 (19.9)			
No/Unknown	1141 (73.9)	257 (63.8)	419 (78.8)	466 (80.1)			
Chemotherapy					<0.001^*^	0.006^*^	0.011^*^
Yes	477 (30.9)	161 (40.0)	129 (24.2)	182 (31.3)			
No/Unknown	1067 (69.1)	242 (60.0)	403 (75.8)	400 (68.7)			

*PUC* Primary urethral carcinoma, *AJCC* American Joint Committee on Cancer

### Survival analyses in the overall patient cohort stratified by surgical procedure

**Fig. 1:**
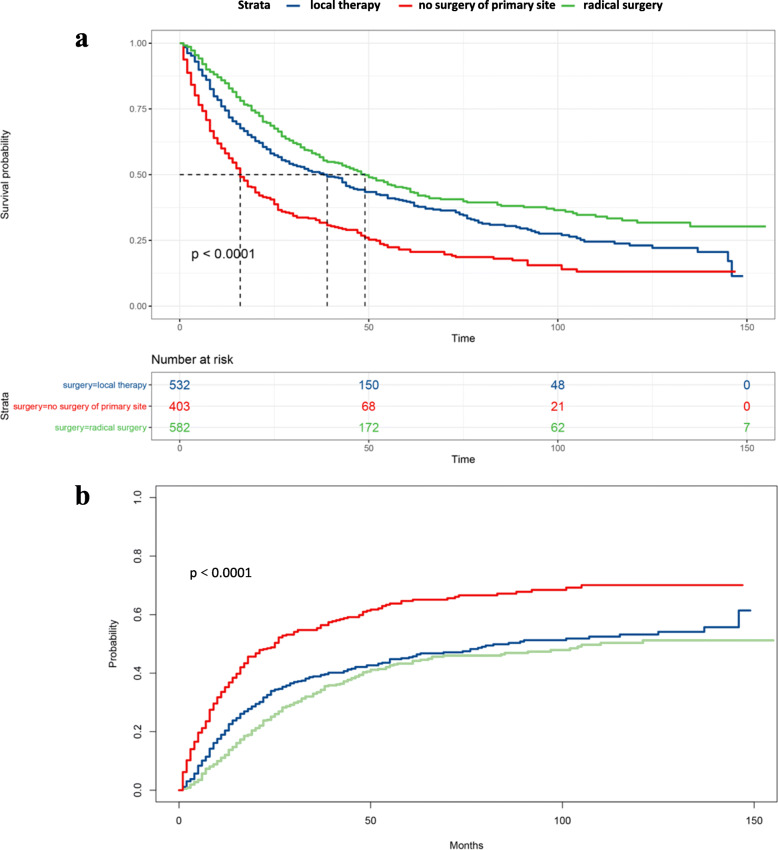
Kaplan-Meier curves and risk tables of OS (**a**), cumulative incidence of CSM (**b**) in PUC patients stratified by surgical procedure

Among the 1544 PUC patients, 403 (26.1%) did not undergo any surgery to the primary tumor, 532 (34.5%) received local therapy (transurethral or transvaginal resection), and 582 (37.7%) underwent radical surgery (urethrectomy). Patient characteristics stratified by surgical procedure are also presented in Table 1. The 5-yr OS was significantly better in patients undergoing either local therapy (39.8%; 95% CI: 35.3–44.7) or radical surgery (44.7%; 95% CI: 40.1–49.7) compared to patients receiving no surgery of the primary site (21.5%; 95% CI: 17.4–26.7) (*p* < 0.001) (Fig. 1 and Table 4). In addition, undergoing local therapy or radical surgery was each independently associated with decreased CSM, with predicted 5-yr cumulative incidence of 45.4 and 43.3%, respectively, compared to 64.7% for patients receiving no surgery of the primary site (*p* < 0.001) (Fig. 1 and Table 4).

### Multivariate cox regression analysis and multivariable competing risks regression analysis

Based on the univariate and multivariate Cox regression model; older age, advanced T stage, lymph node involvement, metastatic disease, and larger tumor size were identified as independent risk factors associated with poorer OS (Table 2). Using a multivariable competing risks regression model, the factors independently associated with increased CSM for PUC patients were identified, included; older age, metastatic disease, advanced AJCC stage groups, and larger tumor size (Table 3). Notably, surgery of the primary site was independently associated with better OS (local therapy, *p* = 0.037; radical surgery, *p* < 0.001) and decreased CSM (*p* = 0.003).

**Table 2:** Univariate and multivariate Cox regression analysis for predicting OS of PUC

	Univariate analysis			Multivariate analysis		
	HR	95%CI	*p* value	HR	95%CI	*p* value
**Age**						
<70	Ref			Ref		
≥ 70	1.906	1.667–2.179	<0.001^*^	1.948	1.693–2.242	<0.001^*^
Sex						
Male	Ref					
Female	0.919	0.803–1.051	0.281			
Race						
White	Ref					
Black	1.102	0.937–1.297	0.241			
Other	1.050	0.786–1.404	0.741			
**Grade**						
I	Ref			Ref		
II	1.192	0.847–1.678	0.313	1.207	0.893–1.632	0.220
III	1.407	1.014–1.953	0.041^*^	1.156	0.862–1.550	0.334
IV	1.342	0.961–1.874	0.085	1.214	0.895–1.646	0.213
**Histology**						
SCC	Ref					
TCC	1.122	0.957–1.314	0.155	0.899	0.749–1.079	0.251
AC	0.906	0.737–1.114	0.301	0.765	0.619–0.946	0.014^*^
Other	1.289	1.037–1.601	0.022^*^	0.898	0.717–1.126	0.352
**T stage**						
T0/T1	Ref			Ref		
T2	1.105	0.913–1.337	0.307	1.125	0.773–1.636	0.539
T3	1.577	1.313–1.894	<0.001^*^	1.444	1.059–1.969	0.020^*^
T4	2.144	1.738–2.646	<0.001^*^	1.788	1.273–2.512	<0.001^*^
**N stage**						
N0	Ref			Ref		
N1	1.530	1.239–1.891	<0.001^*^	1.057	0.837–1.333	0.643
N2	1.810	1.464–2.238	<0.001^*^	1.413	1.073–1.862	0.014^*^
**M stage of BC**						
M0	Ref			Ref		
M1	4.646	3.845–5.615	<0.001^*^	3.080	2.377–3.990	<0.001^*^
**AJCC stage groups**						
I	Ref			Ref		
II	1.075	0.859–1.345	0.528	1.015	0.661–1.558	0.947
III	1.271	1.030–1.570	0.026^*^	1.037	0.722–1.490	0.843
IV	2.685	2.247–3.209	<0.001^*^	0.973	0.658–1.438	0.891
**Tumor size, mm**						
<30	Ref			Ref		
≥ 30	1.662	1.307–2.115	<0.001^*^	1.369	1.176–1.594	<0.001^*^
**Surgery**						
No surgery	Ref			Ref		
Local therapy	0.599	0.511–0.701	<0.001^*^	0.832	0.700–0.989	0.037^*^
Radical therapy	0.458	0.389–0.539	<0.001^*^	0.626	0.524–0.746	<0.001^*^
Radiation						
No/Unknown	Ref					
Yes	1.003	0.866–1.162	0.966			
Chemotherapy						
No/Unknown	Ref					
Yes	0.984	0.855–1.134	0.826			

*OS* Overall survival, *PUC* Primary urethral carcinoma, *HR* Hazard ratio, *CI* Confidence interval, *Ref* Reference, *AJCC* American Joint Committee on Cancer

Grade: Grade I (Well differentiated); Grade II (Moderately differentiated); Grade III (Poorly differentiated); Grade IV (Undifferentiated)

**Table 3:** Multivariable competing risks regression analysis for predicting CSS of PUC

	Multivariable competing risks regression analysis		
	SHR	95%CI	*p* value
**Age**			
<70	Ref		
≥ 70	1.456	1.249–1.698	<0.001^*^
Grade			
I/ II	Ref		
III /IV	1.116	0.939–1.327	0.213
Histology			
SCC/TCC/AC	Ref		
Other	1.008	0.795–1.276	0.950
T stage			
T0/T1/T2	Ref		
T3/T4	1.076	0.811–1.427	0.613
N stage			
N0	Ref		
N1/N2	1.264	0.997–1.603	0.053
**M stage**			
M0	Ref		
M1	2.556	1.986–3.290	<0.001^*^
**AJCC stage groups**			
I/ II	Ref		
III /IV	1.469	1.059–2.039	0.021^*^
**Tumor size, mm**			
<30	Ref		
≥ 30	1.311	1.095–1.568	0.003^*^
**Surgery**			
No surgery	Ref		
Local/Radical therapy	0.760	0.636–0.908	0.003^*^

*CSS* Cancer-specific survival, *PUC* Primary urethral carcinoma, *SHR* Subdistribution hazard ratio

*CI* Confidence interval, *Ref* Reference, *AJCC* American Joint Committee on Cancer

### Subgroup survival analyses based on the risk factors

Subgroup analyses were performed to further evaluate survival benefit of surgery for PUC patients among groups based on age (< 70 vs ≥ 70 years), tumor size (< 30 vs ≥ 30 mm) or sex. Patients who underwent surgery of the primary site showed significant survival advantage in both age subgroups (Supplementary Figure 1 and Table 4). The benefit of surgery was more marked in patients aged < 70 years, with median survival months of 105 and 84 for local therapy and radical surgery, respectively, compared to 21 for patients receiving no surgery of the primary site. Patients who underwent radical surgery exhibited higher OS and decreased CSM regardless of tumor size (Supplementary Figure 2 and Table 4). In contrast there were no significant differences in survival between patients who received local therapy or no surgery of the primary site in either tumor size subgroup. Subset analyses based on sex also revealed that surgery of the primary site brought significant survival benefit regardless of sex (Supplementary Figure 3 and Table 4). To determine whether higher cancer stage affected survival among surgery groups, subset analyses were also performed based on AJCC stage groups. Patients who underwent radical surgery exhibited higher OS and decreased CSM regardless of stage groups (Supplementary Figure 4 and Table 4). Local therapy did not result in significantly greater OS compared to no surgery of the primary site in the I/II stage (*p* = 0.392) or the III/IV stage group (*p* = 0.053), but did result in longer median survival (52 months) compared to no surgery (30 months) in the I/II stage group. Patients who underwent surgery of the primary site showed significant survival advantage in M0 disease, but did not exhibit any benefit in M1 disease (Supplementary Figure 5 and Table 4).

**Table 4:** Subset analyses of survival of PUC patients based on age at diagnosis, AJCC 8th M stage, AJCC 8th stage groups and tumor size and sex

Overall cohort	n	Median Survival (month)	3-yr OS, %	5-yr OS, %	*p* value	CSM, %		
						1-yr	3-yr	5-yr
No surgery of primary site	403	16 (14–20)	32.7 (28.2–38.0)	21.5 (17.4–26.7)	<0.001^* a^	35.2	55.4	64.7
Local therapy	532	39 (29–45)	51.0 (46.7–55.7)	39.8 (35.3–44.7)	0.001^* b^	20.4	38.9	45.4
Radical surgery of primary site	582	49 (42–60)	57.7 (53.4–62.3)	44.7 (40.1–49.7)	<0.001^* c^	12.0	33.5	43.3
Age, yr	n	Median Survival (month)	3-yr OS, %	5-yr OS, %	*p* value	CSM, %		
						1-yr	3-yr	5-yr
<70								
No surgery of primary site	178	21 (16–36)	40.9 (33.9–49.4)	24.7 (18.3–33.4)	<0.001^*^	27.6	53.6	68.9
Local therapy	184	105 (74-NA)	69.3 (62.5–76.7)	58.9 (51.4–67.4)	0.465	11.8	26.4	32.8
Radical surgery of primary site	354	84 (56–117)	65.1 (59.9–70.8)	53.8 (48.1–60.3)	<0.001^*^	10.5	29.2	38.0
≥70								
No surgery of primary site	225	12 (9–17)	26.4 (20.9–33.3)	19.2 (14.3–26.0)	<0.001^*^	41.2	56.8	61.1
Local therapy	348	25 (21–30)	41.6 (36.4–47.4)	30.0 (25.0–35.9)	0.059	24.8	45.3	51.9
Radical surgery of primary site	228	32 (27–38)	45.8 (39.1–53.7)	30.1 (23.7–38.2)	<0.001^*^	14.5	40.4	51.7
AJCC 8th M stage	n	Median Survival (month)	3-yr OS, %	5-yr OS, %	*p* value	CSM, %		
						1-yr	3-yr	5-yr
M0								
No surgery of primary site	258	25 (20–31)	42.2 (36.2–49.1)	29.2 (23.5–36.2)	0.001^*^	23.8	46.2	57.4
Local therapy	451	44 (37–55)	54.8 (50.2–59.9)	42.7 (37.9–48.2)	0.002^*^	15.3	34.0	40.9
Radical surgery of primary site	517	54 (47–63)	61.0 (56.5–65.8)	47.0 (42.2–52.4)	<0.001^*^	9.0	30.0	40.3
M1								
No surgery of primary site	96	7 (5–9)	NA	NA	0.539	66.3	NA	NA
Local therapy	38	7 (5–11)	NA	NA	0.007^*^	71.1	NA	NA
Radical surgery of primary site	22	10 (6–31)	NA	NA	0.040^*^	52.2	NA	NA
AJCC stage groups	n	Median Survival (month)	3-yr OS, %	5-yr OS, %	*p* value	CSM, %		
						1-yr	3-yr	5-yr
I/ II								
No surgery of primary site	106	30 (21–61)	48.4 (39.5–59.3)	39.6 (30.8–51.0)	0.392	19.4	35.9	42.0
Local therapy	331	52 (43–65)	57.9 (52.6–63.8)	46.1 (40.5–52.5)	<0.001^*^	13.3	29.5	35.6
Radical surgery of primary site	215	102 (76-NA)	74.8 (68.7–81.6)	62.5 (55.2–70.6)	<0.001^*^	2.0	14.7	24.7
III /IV								
No surgery of primary site	206	14 (12–16)	25.2 (19.5–36.7)	12.9 (8.3–19.9)	0.053	44.6	68.1	79.7
Local therapy	126	16 (11–23)	31.8 (24.0–42.0)	21.7 (17.4–32.0)	<0.001^*^	38.6	62.1	69.7
Radical surgery of primary site	315	35 (28–45)	48.3 (42.5–54.8)	34.3 (28.5–41.4)	<0.001^*^	16.7	43.6	53.6
Tumor size, mm	n	Median Survival (month)	3-yr OS, %	5-yr OS, %	*p* value	CSM, %		
						1-yr	3-yr	5-yr
<30								
No surgery of primary site	32	44 (28-NA)	60.4 (44.2–82.4)	34.5 (16.8–70.6)	0.340	19.5	35.5	61.4
Local therapy	60	69 (35-NA)	61.9 (49.6–77.2)	52.7 (40.2–69.2)	0.086	9.1	26.2	30.8
Radical surgery of primary site	136	NA	71.3 (63.2–80.4)	58.8 (49.4–70.1)	0.032^*^	3.9	19.3	30.1
≥30								
No surgery of primary site	92	16 (12–25)	29.7 (21.1–41.8)	18.7 (11.4–30.5)	0.366	39.3	62.7	72.1
Local therapy	62	19 (12–38)	36.4 (25.4–52.3)	21.9 (12.6–37.9)	0.005^*^	30.8	53.2	60.5
Radical surgery of primary site	252	37 (30–48)	50.0 (43.7–57.3)	37.7 (31.2–45.4)	<0.001^*^	16.8	42.5	52.2
Sex	n	Median Survival (month)	3-yr OS, %	5-yr OS, %	*p* value	CSM, %		
						1-yr	3-yr	5-yr
Male								
No surgery of primary site	233	16 (12–20)	32.7 (26.8–39.8)	23.2 (17.8–30.2)	<0.001^*^	39.3	56.6	63.5
Local therapy	385	38 (28–46)	51.1 (46.1–56.6)	39.1 (34.0–45.0)	0.025^*^	21.4	38.8	45.0
Radical surgery of primary site	333	46 (37–60)	55.8 (50.1–62.1)	42.5 (36.5–49.5)	<0.001^*^	13.1	33.4	42.8
Female								
No surgery of primary site	170	16 (15–25)	32.8 (26.1–41.2)	19.0 (13.2–27.5)	<0.001^*^	29.7	53.8	66.7
Local therapy	147	39 (25–76)	50.7 (42.5–60.5)	41.3 (33.1–51.6)	0.045^*^	17.6	39.4	46.8
Radical surgery of primary site	249	52 (44–84)	60.1 (53.9–67.1)	47.5 (40.9–55.1)	<0.001^*^	10.6	33.6	43.7

*PUC* Primary urethral carcinoma, *AJCC* American Joint Committee on Cancer, *OS* Overall survival, *CSM* Cancer-specific mortality

^**a**^ comparing survival of patients with no surgery of primary site to patients with local therapy

^**b**^ comparing survival of patients with local therapy to patients with radical surgery

^**c**^ comparing survival of patients with no surgery of primary site to patients with radical surgery

### Cox’s and competing risks’ proportional hazard analyses

**Fig. 2:**
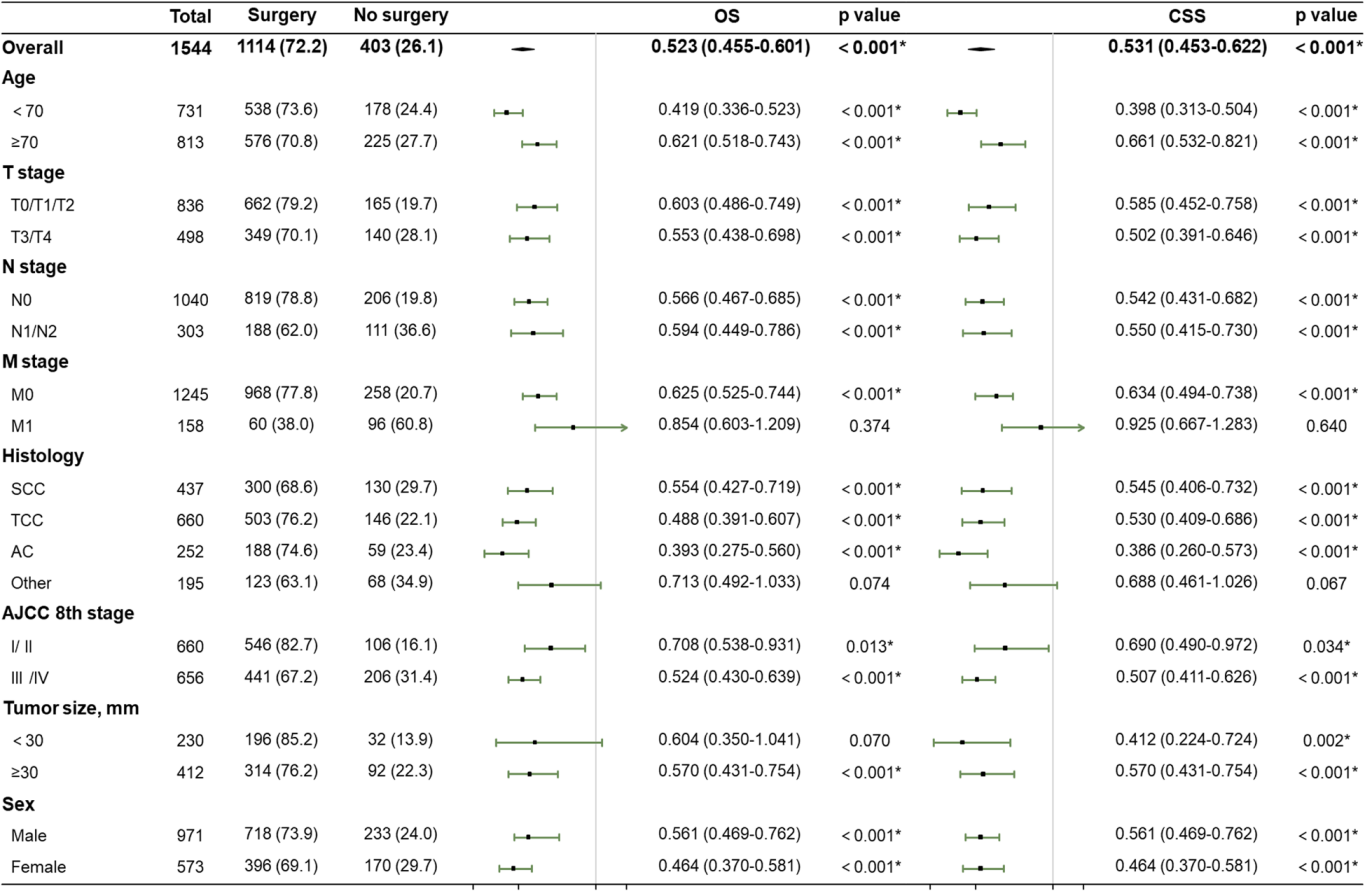
Forest plots summarizing the HRs and 95% CIs of OS and CSS in PUC patients stratified by surgical procedure

Finally, Cox’s and competing risks’ proportional hazard analyses were performed to assess the prognostic value of surgery in PUC patients (Fig. 2). Surgery of the primary site independently predicted statistically significantly higher OS and CSS in both age group (< 70 years, OS: *p* < 0.001, CSS: *p* < 0.001; ≥ 70 years, OS: *p* < 0.001, CSS: *p* < 0.001), both AJCC T stages (T0/T1/T2, OS: *p* < 0.001, CSS: *p* < 0.001; T3/T4, OS: *p* < 0.001, CSS: *p* < 0.001), both AJCC N stages (N0, OS: *p* < 0.001, CSS: *p* < 0.001; N1/N2, OS: *p* < 0.001, CSS: *p* < 0.001), both AJCC stage groups (I/ II, OS: *p* = 0.013, CSS: *p* < 0.001; III /IV, OS: *p* < 0.001, CSS: *p* = 0.034), both sexes (male, OS: *p* < 0.001, CSS: *p* < 0.001; female, OS: *p* < 0.001, CSS: *p* < 0.001), the larger tumor size group (OS: *p* < 0.001, CSS: *p* < 0.001), and the M0 group (OS: *p* < 0.001, CSS: *p* < 0.001), but surgery of the primary site was not an independent risk factor in the M1 group (OS: *p* = 0.374, CSS: *p* = 0.640) or the other histology group (OS: *p* = 0.074, CSS: *p* = 0.067). Notably, surgery of the primary site was an independent risk factor in the smaller tumor size group based only on the competing risks’ proportional hazard analyses (*p* = 0.002).

## Discussion

PUC is an aggressive and rare carcinoma, comprising < 1% of all genitourinary malignancies [7, 13]. The disease management of PUC requires multimodal therapy to improve functional outcome and quality of life. According to the National Comprehensive Cancer Network (NCCN) and European Association of Urology (EAU) guidelines, partial urethrectomy or urethra-sparing surgery is a valid treatment option for localized distal tumors (I/II stage), and Ta-Tis-T1 PUC can also be treated with a repeat transurethral or transvaginal resection. For patients with locally advanced disease (III/IV stage), multimodal treatment strategies are needed to optimize local control and prognosis. Chemotherapy followed by surgery or radiation therapy and concurrent chemoradiation with or without surgery have been shown to lead to an improvement in survival [2, 7, 14, 15]. However, given the rarity of PUC, there are few prospective multi-institutional studies to compare the effectiveness of various multimodal therapies, and the role of surgery in the management of PUC remains contentious.

To our knowledge, this is the first large population-based study to investigate the benefit of surgery for PUC patients. Our results demonstrated that PUC patients who underwent radical surgery or local therapy had a higher 5-yr OS and decreased CSM compared with patients who did not receive surgery of the primary site. Subgroup analysis based on TNM stage also demonstrated that survival of PUC patients who underwent surgery of the primary site was improved regardless of T stage, N stage, or AJCC prognostic group. In terms of M stage, PUC patients with metastatic disease were less likely to benefit from surgery. Notably, PUC patients with early TNM stage (I/II) who received radical surgery showed a more marked survival benefit, indicating that these patients were optimal candidates for urethrectomy.

We noted that factors independently associated with poor OS and increased CSM in PUC patients other than advanced TNM stage included age ≥ 70 years and tumor size ≥30 mm. Subset analyses revealed that patients < 70 years and tumor size ≥30 mm had a notably better survival benefit from surgery. Nevertheless, surgery of the primary site independently predicted significantly better prognosis in both age subgroups. Several studies have demonstrated anatomic differences between male and female PUC patients that contribute to variations in clinicopathological characteristics, including tumor location and histology [10, 16, 17]. In contrast we did not observe any difference in survival between males and females, and surgery of the primary site independently predicted statistically significantly higher OS and decreased CSM in both male and female PUC patients. SCC, TCC and AC together comprised approximately 90% of the histological types of PUC, and previous studies have demonstrated poorer survival in rare PUC pathological types [2, 18]. Subset analyses in our study demonstrated that PUC patients with the predominant histological types who underwent surgery of the primary site showed a significant survival advantage, while PUC patients with rare histological types were less likely to benefit from surgery. The results underscore the continued importance of improved guidelines for management of patients with rare PUC pathological types.

Despite several promising results, this registry-based study has unavoidable limitations. First, limitations of SEER-based studies included the absence of detail with regard to individual information about chemotherapy regimen and radiotherapy doses/fields. Thus, it was not possible to examine the effects of combined surgery and chemotherapy or radiotherapy on patient survival. Second, SEER also lacked information regarding the location of PUC, a significant prognostic factor that undoubtedly influences the treatment strategy. Moreover, this study is a retrospective analysis, and selection bias could not be completely avoided.

## Conclusion

Despite the limitations of this study, our results suggest that surgery for the primary tumor conferred a survival advantage in non-metastatic PUC patients regardless of sex, age, T stage, and N stage. Furthermore, the surgical benefit was more marked in patients with early TNM stage (I/II) disease, patients < 70 years, and those with tumor size ≥30 mm.

## Supplementary Information

**Additional file 1: Supplementary Figure 1.** OS and CSM in PUC patients stratified by surgical procedure and age. **(a)** Patients aged < 70 years, **(b)** patients aged ≥70 years.

**Additional file 2: Supplementary Figure 2.** OS and CSM in PUC patients stratified by surgical procedure and tumor size. **(a)** Tumor size < 30 mm, **(b)** tumor size ≥30 mm.

**Additional file 3: Supplementary Figure 3.** OS and CSM in PUC patients stratified by surgical procedure and sex. **(a)** Male, **(b)** female.

**Additional file 4: Supplementary Figure 4.** OS and CSM in PUC patients stratified by surgical procedure and AJCC stage groups. **(a)** I/II stage, **(b)** III/IV stage.

**Additional file 5: Supplementary Figure 5.** OS and CSM in PUC patients stratified by surgical procedure and M stage. **(a)** Stage M0, **(b)** stage M1.

## Data Availability

The data that support the findings of this study are openly available in the Surveillance, Epidemiology and End Results (SEER) database of the National Cancer Institute.

## Abbreviations

PUC: Primary urethral carcinoma
OS: Overall survival
CSM: Cancer-specific mortality
CSS: Cancer-specific survival
SEER: The Surveillance, Epidemiology and End Results database
ICD-O-3: The International Classification of Diseases-O-3
AJCC: American Joint Committee on Cancer
HR: Hazard ratio
CI: Confidence interval
Ref: Reference
