# Supplementary figures and images for: Primary tumor surgery improves survival in non-metastatic primary urethral carcinoma patients: a large population-based investigation
Source: BMC Cancer. 2021 Jul 27;21:857. doi: 10.1186/s12885-021-08603-z (PMC8314574; doi:10.1186/s12885-021-08603-z)

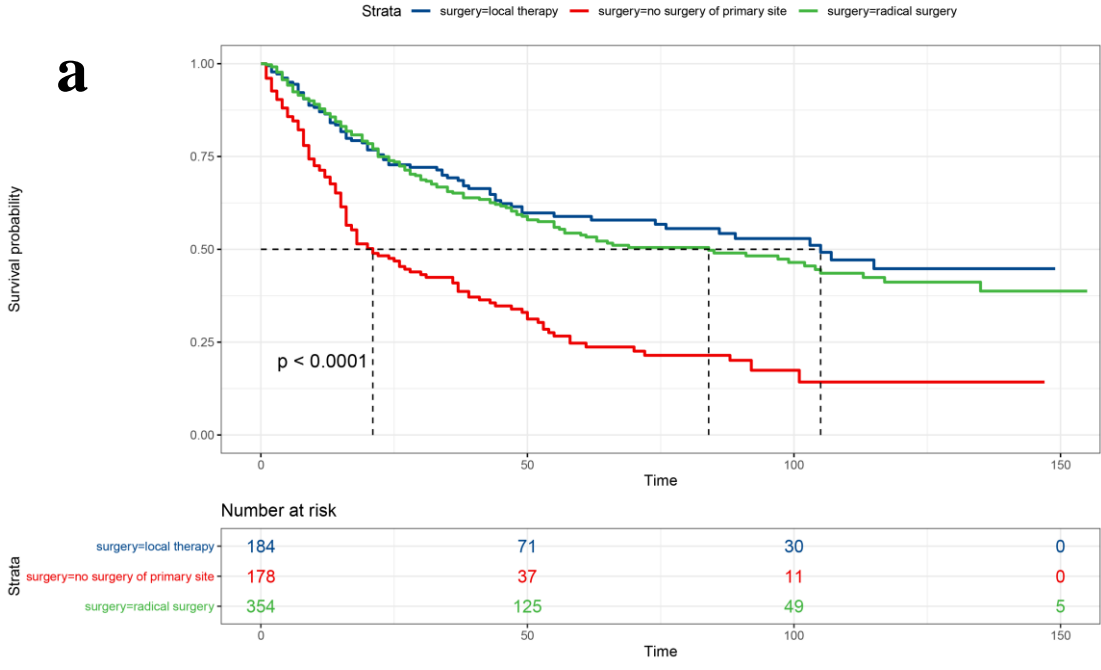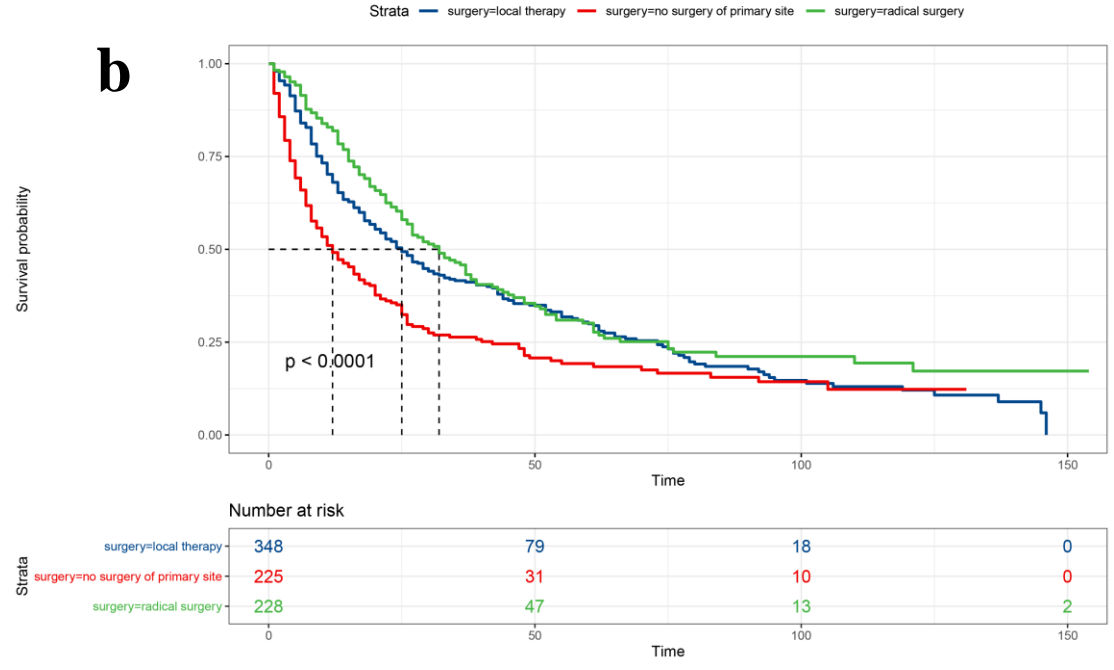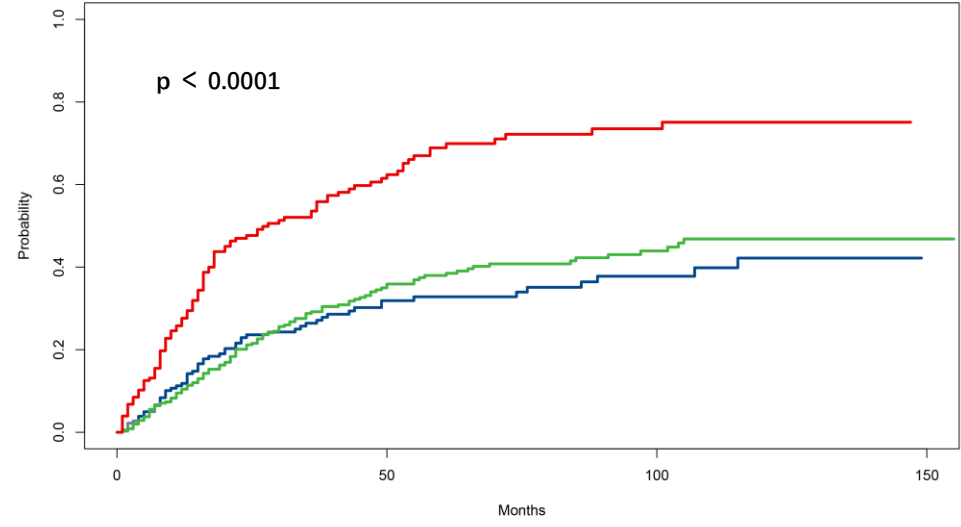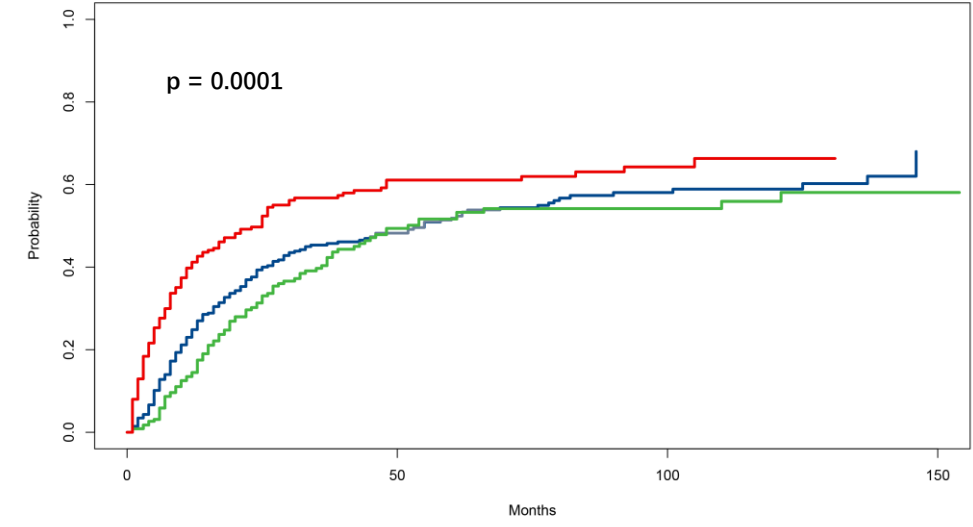

Supplement: Supplementary file 1 — Additional file 1: Supplementary Figure 1. OS and CSM in PUC patients stratified by surgical procedure and age. (a) Patients aged < 70 years, (b) patients aged ≥70 years. [file 12885_2021_8603_MOESM1_ESM.pdf]

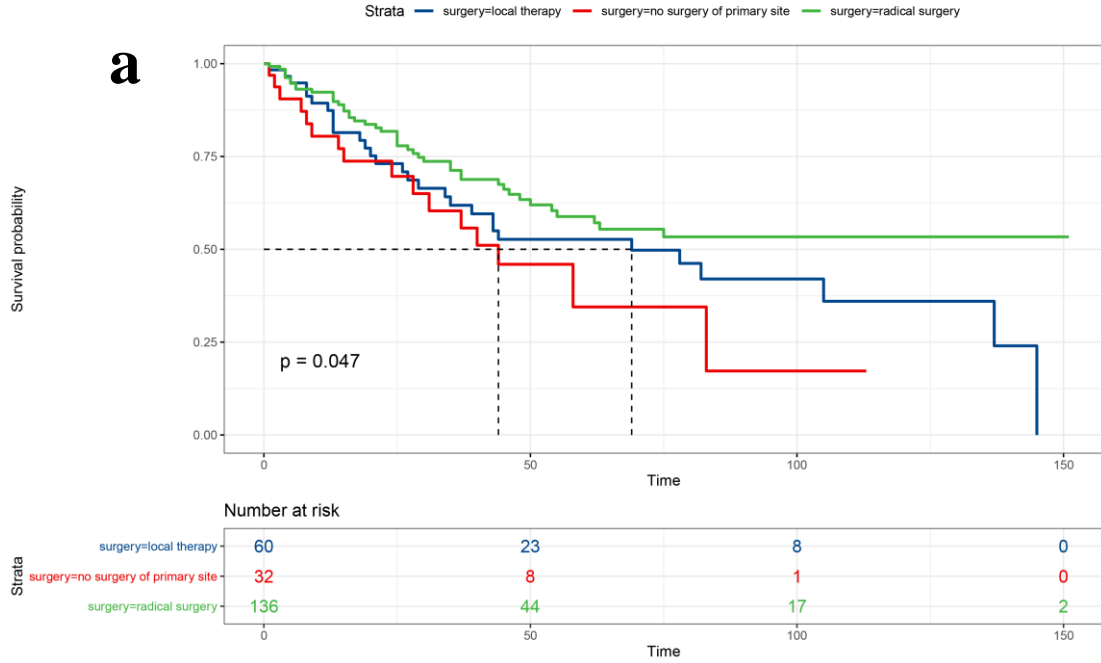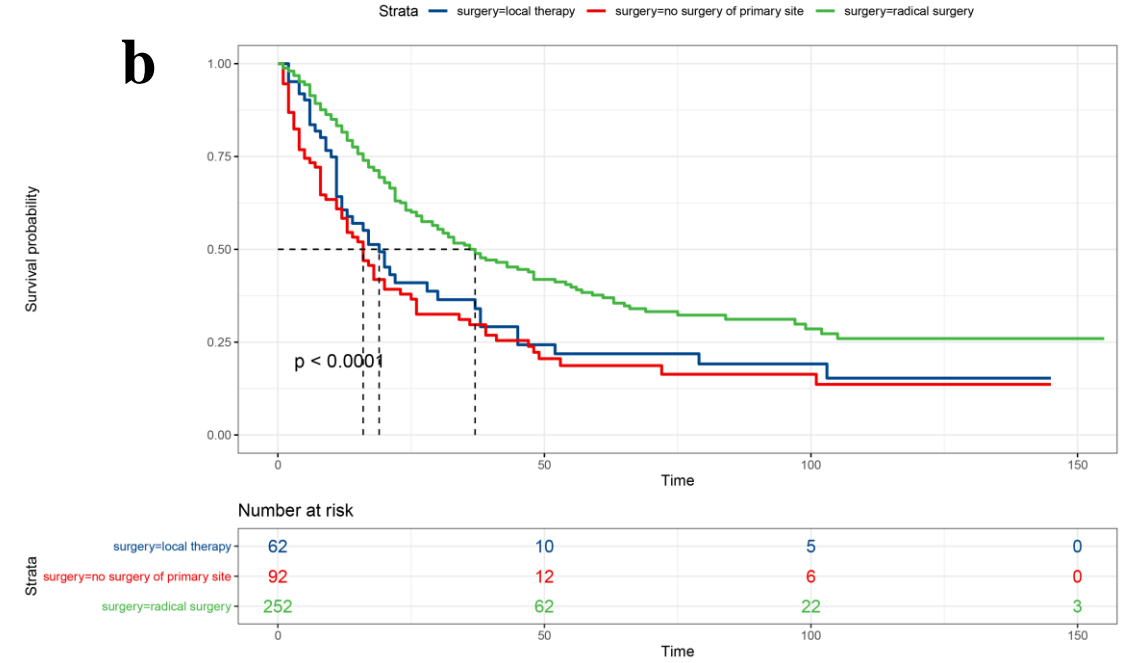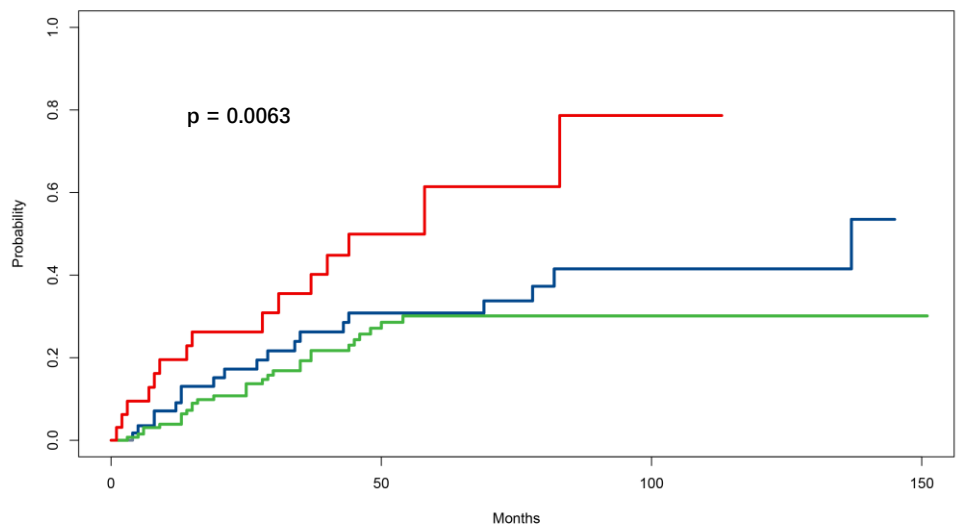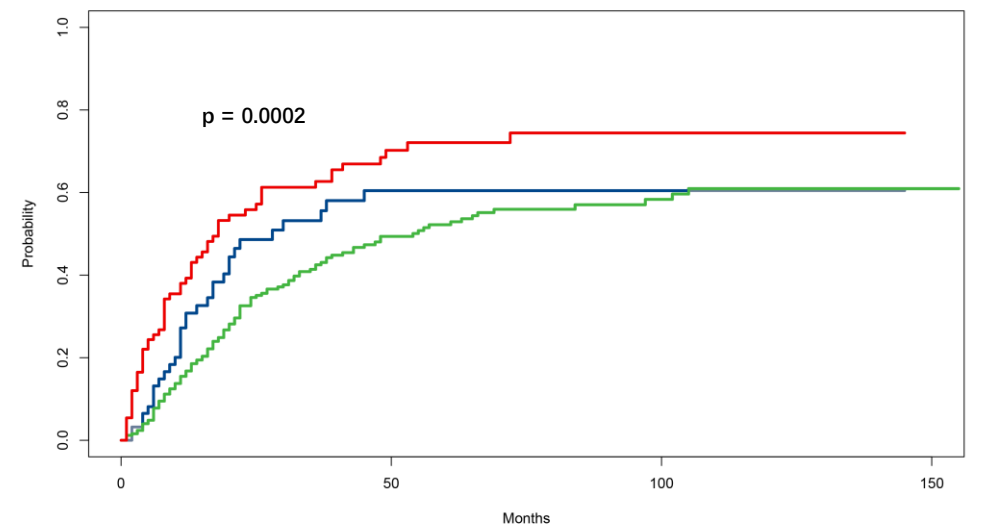

Supplement: Supplementary file 2 — Additional file 2: Supplementary Figure 2. OS and CSM in PUC patients stratified by surgical procedure and tumor size. (a) Tumor size < 30 mm, (b) tumor size ≥30 mm. [file 12885_2021_8603_MOESM2_ESM.pdf]

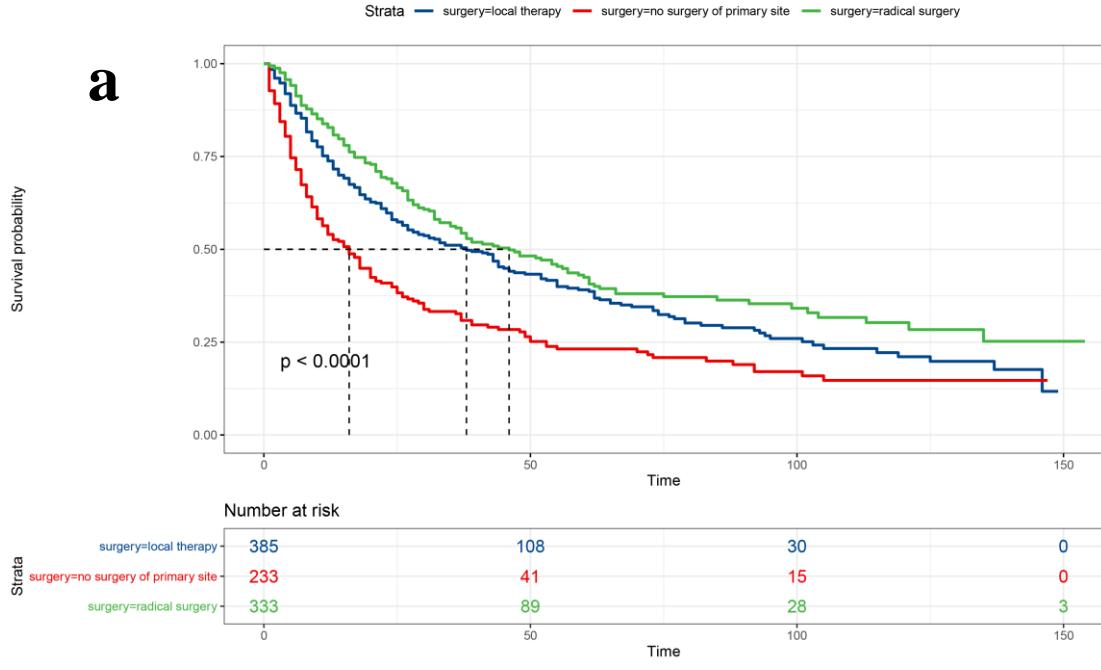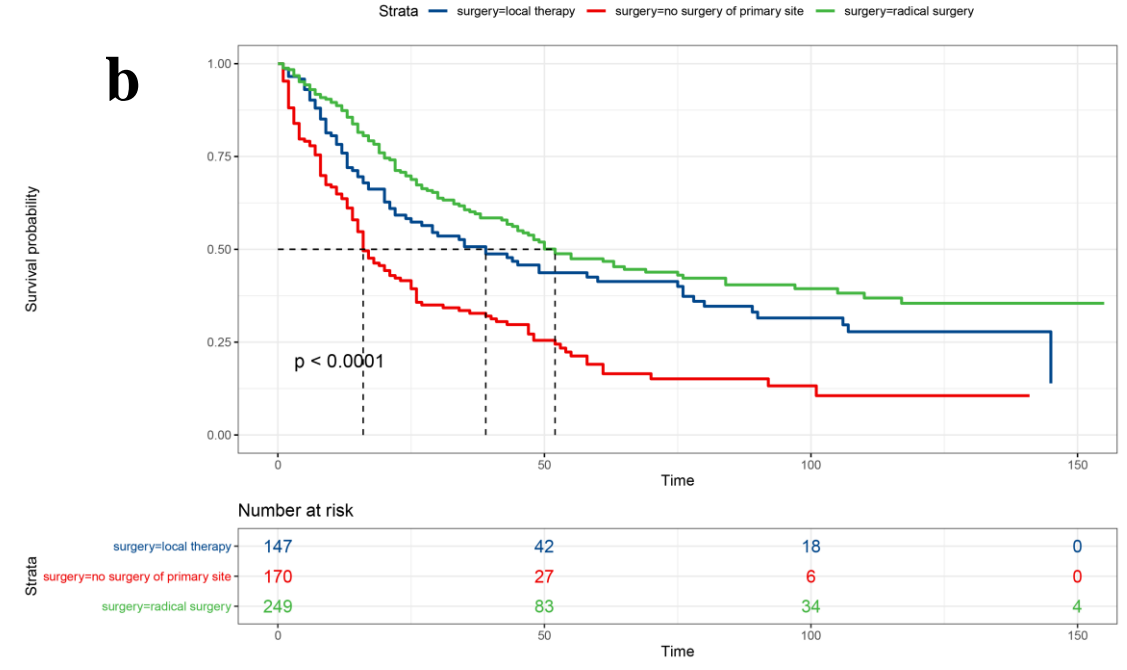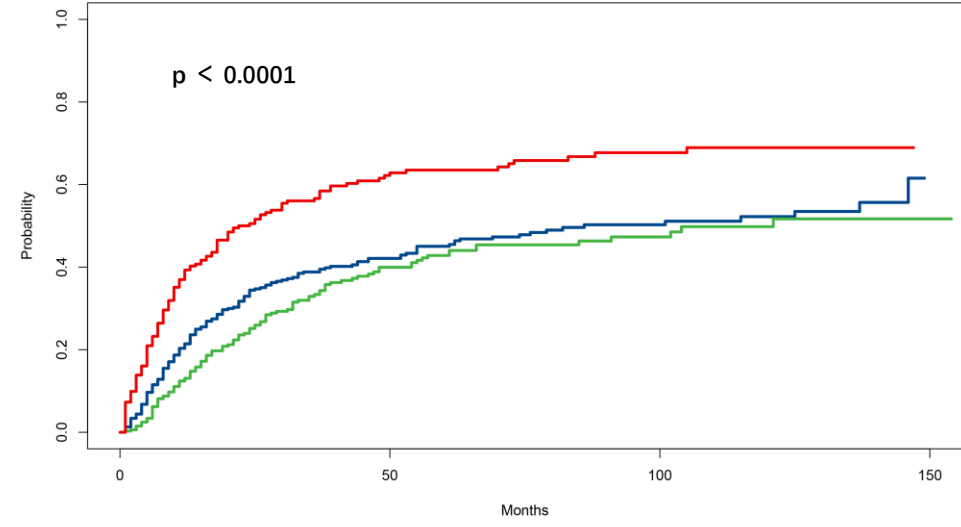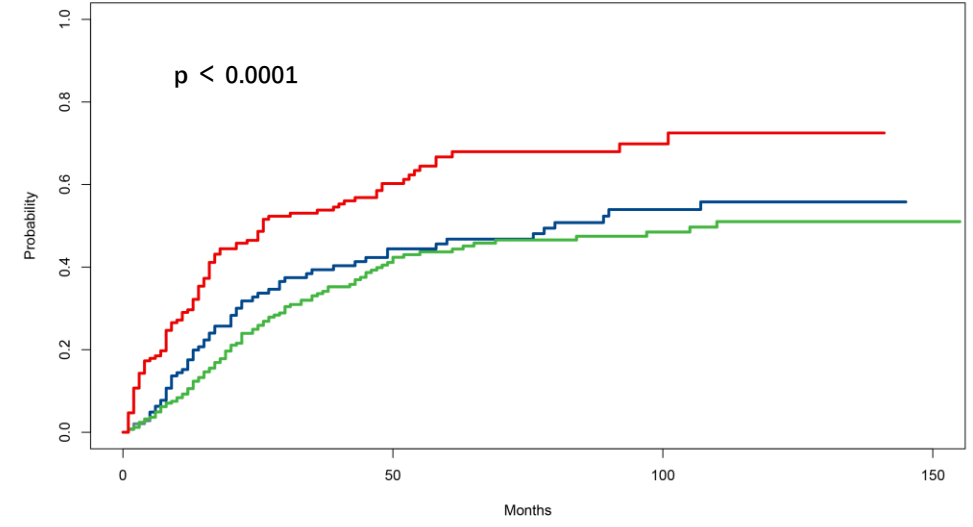

Supplement: Supplementary file 3 — Additional file 3: Supplementary Figure 3. OS and CSM in PUC patients stratified by surgical procedure and sex. (a) Male, (b) female. [file 12885_2021_8603_MOESM3_ESM.pdf]

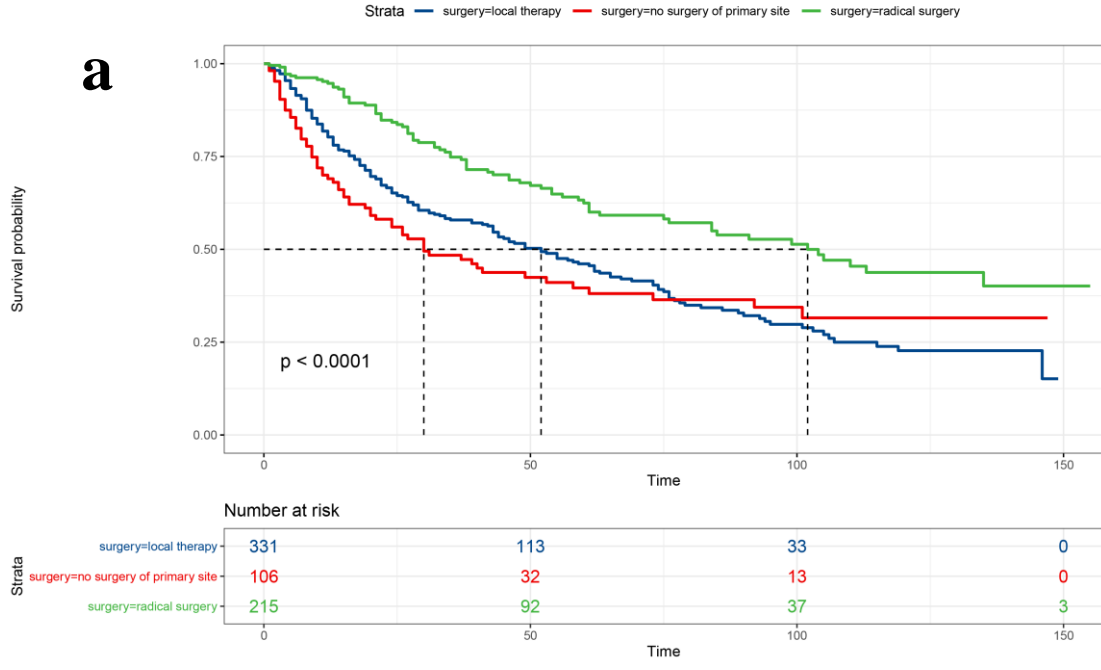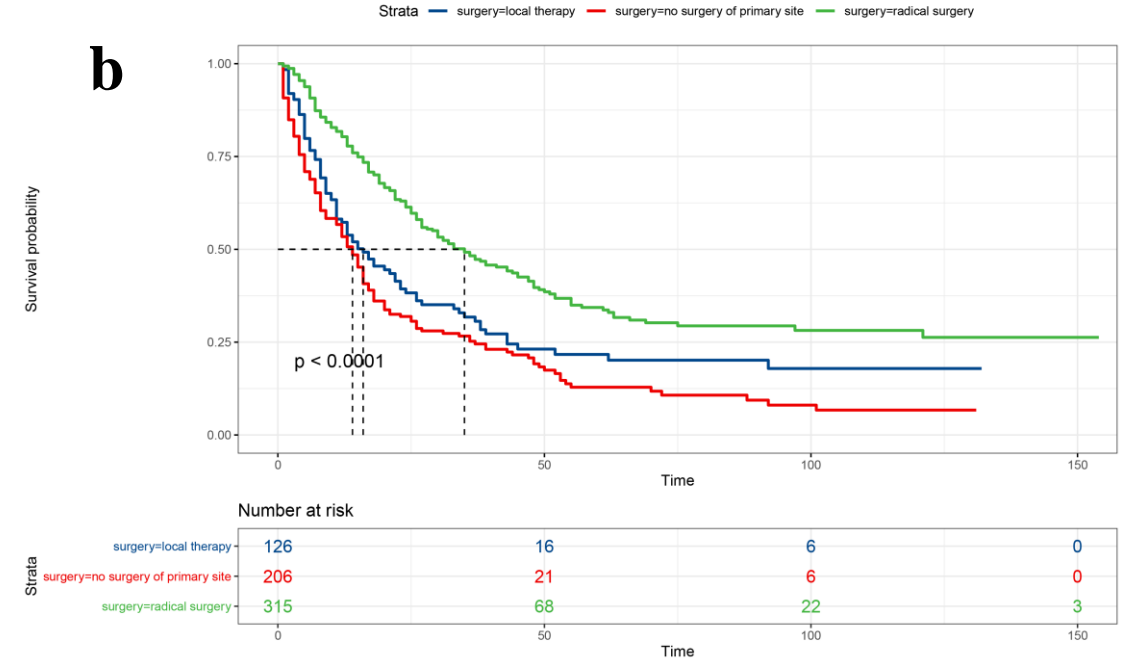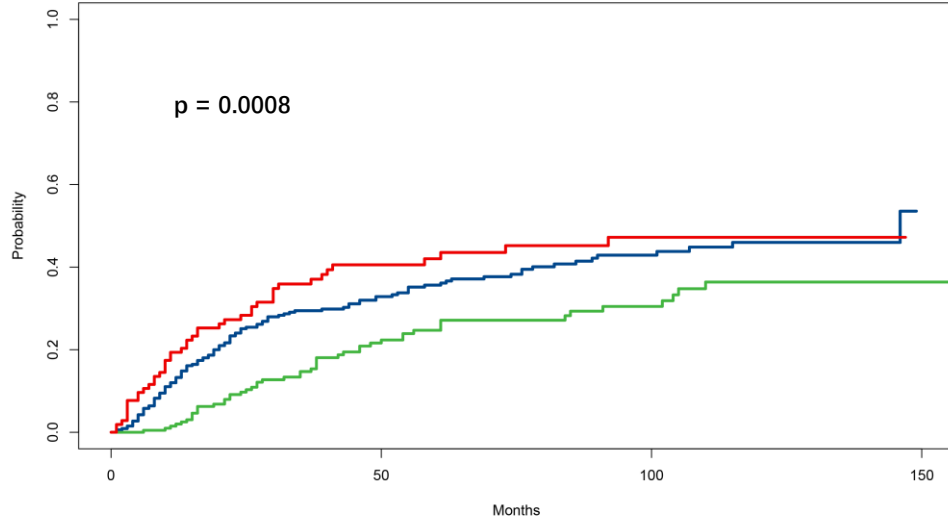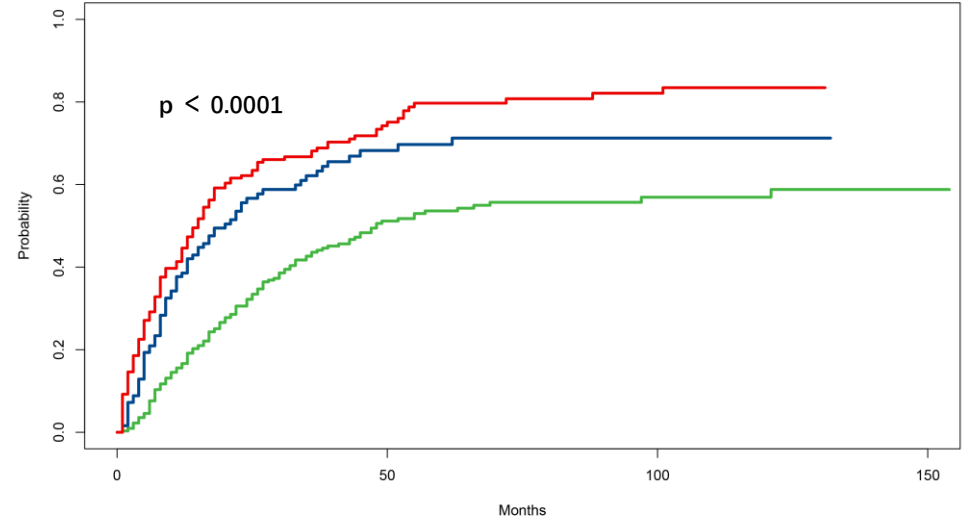

Supplement: Supplementary file 4 — Additional file 4: Supplementary Figure 4. OS and CSM in PUC patients stratified by surgical procedure and AJCC stage groups. (a) I/II stage, (b) III/IV stage. [file 12885_2021_8603_MOESM4_ESM.pdf]

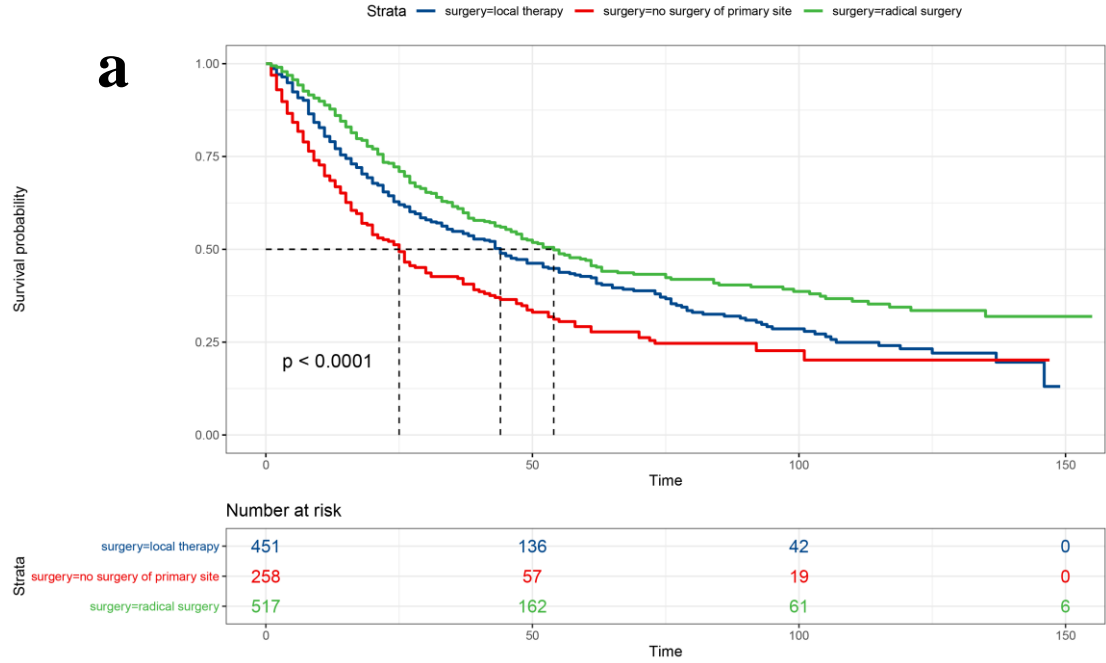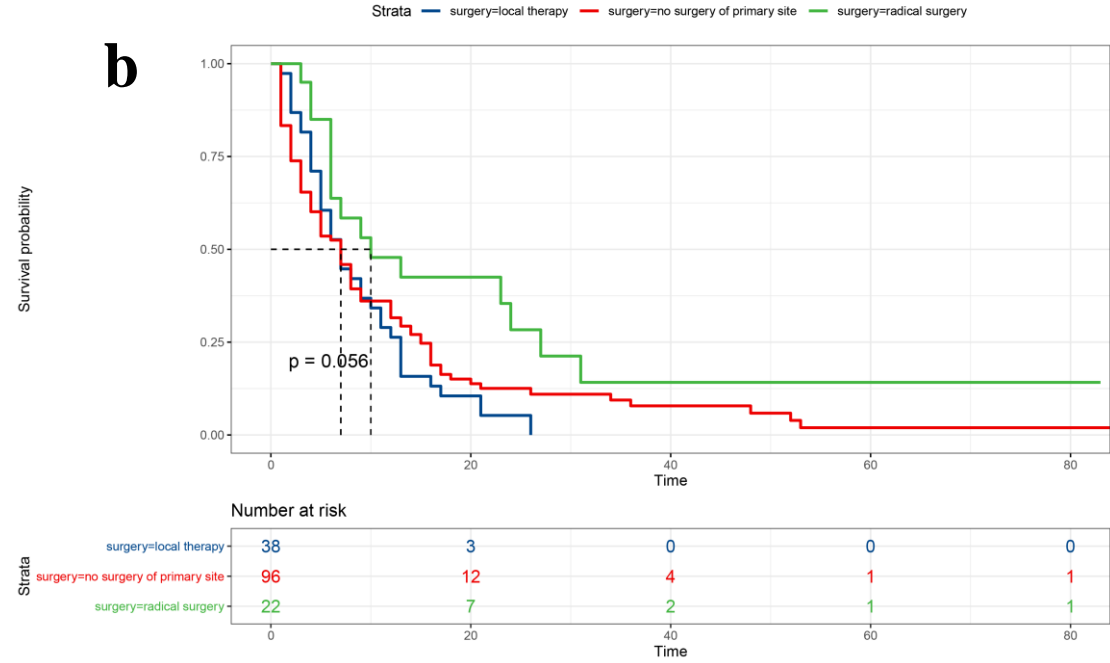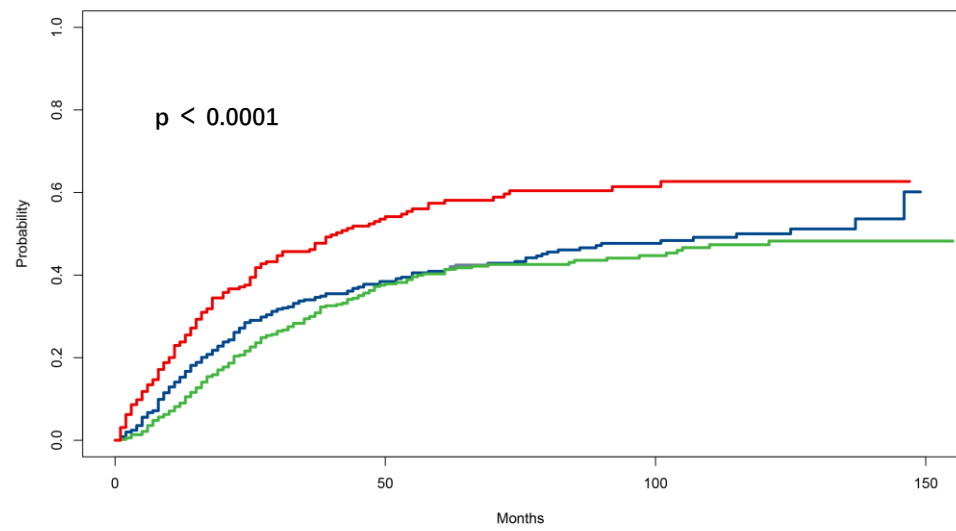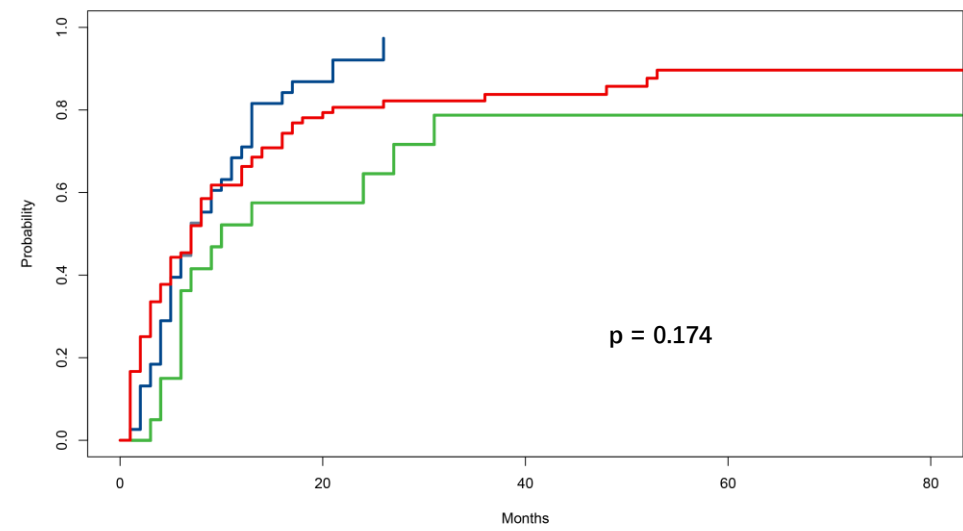

Supplement: Supplementary file 5 — Additional file 5: Supplementary Figure 5. OS and CSM in PUC patients stratified by surgical procedure and M stage. (a) Stage M0, (b) stage M1. [file 12885_2021_8603_MOESM5_ESM.pdf]
